# Comparison of Toxicities among Different Bumped Kinase Inhibitor Analogs for Treatment of Cryptosporidiosis

**DOI:** 10.1128/aac.01425-22

**Published:** 2023-03-15

**Authors:** Matthew A. Hulverson, Ryan Choi, Deborah A. Schaefer, Dana P. Betzer, Molly C. McCloskey, Grant R. Whitman, Wenlin Huang, Sangun Lee, Andy Pranata, Malcolm D. McLeod, Kennan C. Marsh, Dale J. Kempf, Bruce E. LeRoy, Mark T. Zafiratos, Aimee L. Bielinski, Robert C. Hackman, Kayode K. Ojo, Samuel L. M. Arnold, Lynn K. Barrett, Saul Tzipori, Michael W. Riggs, Erkang Fan, Wesley C. Van Voorhis

**Affiliations:** a Department of Medicine, Division of Allergy and Infectious Disease, Center for Emerging and Reemerging Infectious Disease (CERID), University of Washington, Seattle, Washington, USA; b School of Animal and Comparative Biomedical Sciences, College of Agriculture and Life Sciences, University of Arizona, Tucson, Arizona, USA; c Department of Biochemistry, University of Washington, Seattle, Washington, USA; d Department of Infectious Disease and Global Health, Cummings School of Veterinary Medicine at Tufts University, North Grafton, Massachusetts, USA; e Research School of Chemistry, Australian National University, Canberra, ACT, Australia; f Research and Development, AbbVie, Inc., North Chicago, Illinois, USA; g Former employee of AbbVie, Inc., North Chicago, Illinois, USA; h Fred Hutchinson Cancer Research Center, Seattle, Washington, USA; i Department of Pathology, University of Washington, Seattle, Washington, USA; j Department of Laboratory Medicine, University of Washington, Seattle, Washington, USA

**Keywords:** BKI-1708, BKI-1770, BKI-1841, *Cryptosporidium*, bumped kinase inhibitors, calcium-dependent protein kinases, cryptosporidiosis, epiphyseal growth plate

## Abstract

Recent advances on the development of bumped kinase inhibitors for treatment of cryptosporidiosis have focused on the 5-aminopyrazole-4-carboxamide scaffold, due to analogs that have less hERG inhibition, superior efficacy, and strong *in vitro* safety profiles. Three compounds, BKI-1770, -1841, and -1708, showed strong efficacy in C. parvum infected mice. Both BKI-1770 and BKI-1841 had efficacy in the C. parvum newborn calf model, reducing diarrhea and oocyst excretion. However, both compounds caused hyperflexion of the limbs seen as dropped pasterns. Toxicity experiments in rats and calves dosed with BKI-1770 showed enlargement of the epiphyseal growth plate at doses only slightly higher than the efficacious dose. Mice were used as a screen to check for bone toxicity, by changes to the tibia epiphyseal growth plate, or neurological causes, by use of a locomotor activity box. These results showed neurological effects from both BKI-1770 and BKI-1841 and bone toxicity in mice from BKI-1770, indicating one or both effects may be contributing to toxicity. However, BKI-1708 remains a viable treatment candidate for further evaluation as it showed no signs of bone toxicity or neurological effects in mice.

## INTRODUCTION

*Cryptosporidium* is a leading cause of diarrheal disease among children under 2 years old within the resource limited areas of the world, including southeast Asia and sub-Saharan Africa (1). The Global Burden of Diseases, Injuries, and Risk Factors Study, conducted in 2016, determined that *Cryptosporidium* was responsible for over 48,000 deaths and 4.2 disability-adjusted life-years lost to children under the age of 5 (2). The only clinically approved treatment for *Cryptosporidium*, Nitazoxanide, has limited efficacy in healthy individuals, is not approved for use in infants, is only about 30% efficacious in malnourished children (subtracting placebo efficacy), and does not clear infections in immunocompromised individuals, where infection becomes chronic and often fatal (3–5). Progress in drug development research has been advanced in recent years for a number of possible drug targets through new techniques in compound screening and genetic manipulation of the parasite (6–9).

Bumped kinase inhibitors (BKIs) targeting calcium-dependent protein kinase 1 (CDPK1) have proven to be effective against apicomplexan parasites in several *in vitro* and *in vivo* laboratory models (10–15). CDPK1 is expressed during the asexual stages of the life cycle, playing a role in intestinal epithelium invasion and egress of the parasite and is essential to parasite viability (16). Many BKI analogs, containing the central scaffolds pyrazolopyrimidine (PP) or 5-aminopyrazole-4-carboxamide (AC), have shown promising efficacy results in Cryptosporidium parvum infected mice (6, 17, 18), C. hominis infected gnotobiotic piglets (12), and C. parvum infected neonatal calves (13, 18).

Among these analogs, some issues have arisen that could preclude human clinical treatment due to toxicity. The first issue identified was potential cardiotoxicity as indicated in BKI-1294, a PP analog with inhibition of the human ether-à-go-go-related gene (hERG) in the submicromolar range *in vitro* (19). Cardiotoxicity was additionally seen in PP BKI-1369 in the *in vitro* hERG assay as well as *in vivo* with cardiotoxicity testing in rats (18) and dogs (data not shown).

Medicinal chemistry efforts around the AC scaffold of BKIs has yielded several analogs that circumvent potential cardiotoxicity issues (17). However, additional liabilities arose in several of these compounds, including systemic and gastrointestinal (GI) toxicities, teratogenicity, and loss of efficacy against the target parasite (20). In addition to these liabilities, chirality presented the potential obstacle of raising the production cost of a treatment needed primarily in resource limited regions of the world (20, 21).

From these ongoing efforts, several promising candidates emerged, particularly among the AC scaffold series. BKIs -1770 and -1708 demonstrated promising *in vivo* efficacy in Nanoluciferase (Nluc) expressing C. parvum infected Interferon-γ knockout (IFN-γ KO) mice (17). Additionally, both the BKI-1841 and -1770 chemical structures were designed to possess achiral substituent groups as alternatives to other efficacious, yet chiral, BKIs (20), and BKI-1770 and -1708 showed no signs of cardiotoxicity in rats or dogs (20). With these BKIs showing efficacy and a lack of toxicity in rodents, the next progression in evaluating them as treatment candidates was evaluation in large animals, namely, neonatal calves (13), and further toxicity studies involving multiple and escalated dosing. These results are essential, since variation of the substituent groups have been shown to greatly affect the toxicity observed, even among different molecules sharing the same central scaffold (6, 17, 18, 20, 22, 23). BKI-1770 was prioritized for efficacy against C. parvum in neonatal calves and toxicity testing in rats and calves, with follow-up efficacy testing in calves for BKI-1841 and additional toxicity screening in mice for BKI-1770, -1841, and -1708.

## RESULTS

### *In vitro* screening.

**FIG 1:**
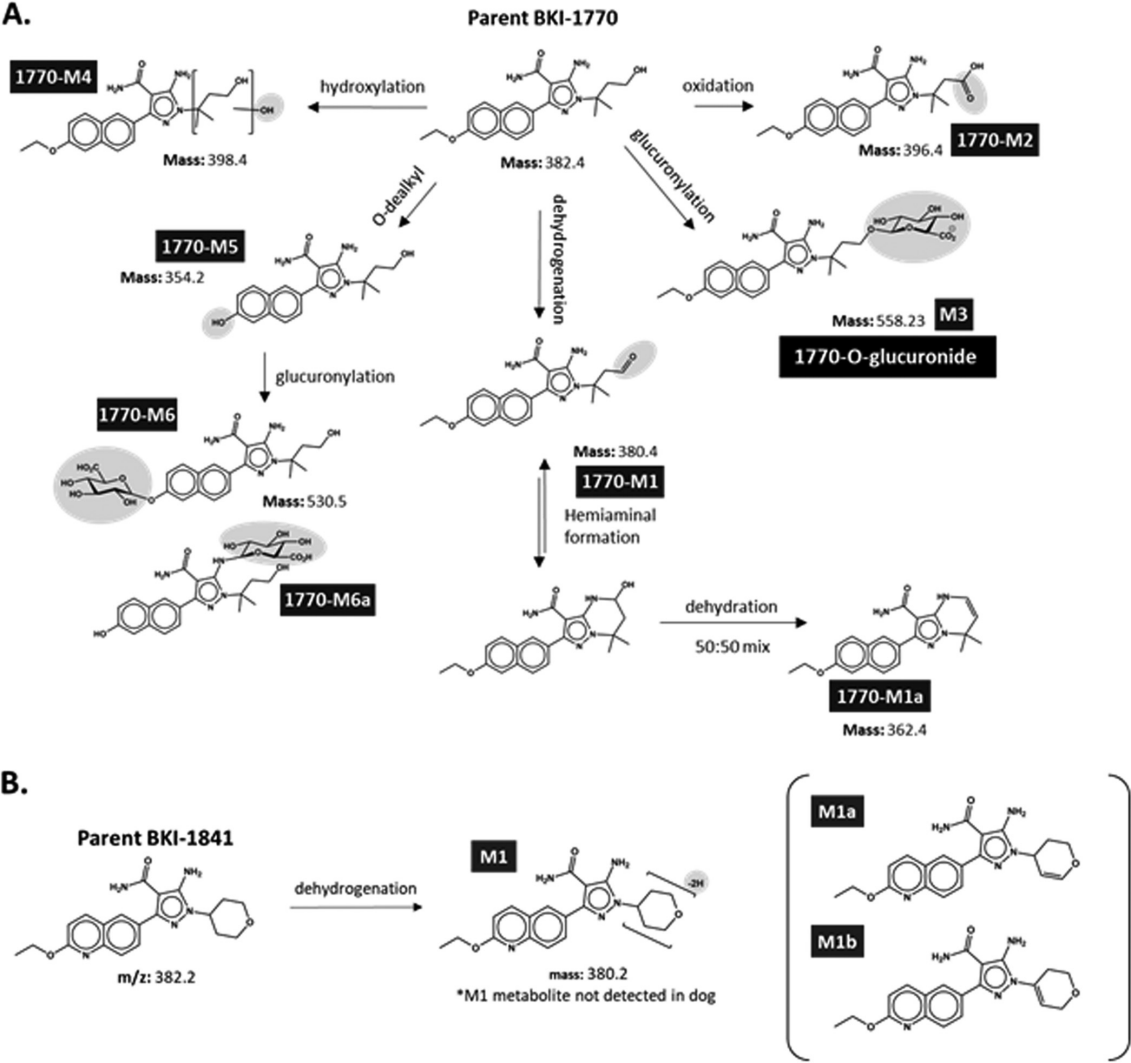
(A) Structures of probable metabolites of BKI-1770 identified by hepatocyte metabolism *in vitro*. For BKI-1770, corresponding ions for three metabolites were observed when *in vivo* mouse plasma samples were extracted and scanned on LC/MS-MS. Metabolite 1 (M1) was synthesized for quantification purposes. This stock of M1 was estimated to be a 50:50 mix of the hemiaminal (*m/z*: 380.4) and its dehydrated product (*m/z*: 362.4) labeled M1a. Metabolite 3 (M3) was synthesized as 1770-O-glucuronide. Metabolite 5 (M5) was synthesized for quantification purposes. No corresponding *m/z* signals were observed in mouse plasma on the LC/MS-MS scan for metabolite 2 (M2), metabolite 4 (M4), or metabolite 6 (M6) or 6a (M6a). (B) Structures of probable metabolites of BKI-1841 identified by hepatic S9 fraction metabolism *in vitro*. For BKI-1841, no corresponding ions for metabolite 1 (M1), metabolite M1a (M1a), or metabolite M1b (M1b) were observed when *in vivo* mouse plasma samples were extracted and scanned on LC/MS-MS. *m/z* = mass to ion ratio.

BKI-1770 was previously screened for *in vitro* inhibition of *Cp*CDPK1 and *Hs*SRC, cytotoxicity against HepG2 and CRL-8155 cells, hERG liability, and aqueous solubility (17, 23) (Table 1). BKI-1841 was also evaluated in these assays (Table 1). All results from these assays were favorable, including falling within the solubility range that correlated with efficacy for the AC scaffold (17). Further *in vitro* characterization was performed on both compounds, namely, the determination of probable metabolites for BKI-1770 using hepatocytes and for BKI-1841 using hepatic S9 fractions from mice, rats, dogs, monkeys, and humans (Fig. 1, Fig. S2).

**TABLE 1:** *In vitro* properties of bumped kinase inhibitors^*a*^

BKI	*Cp*CDPK1 IC_50_ (μM)	*C. parvum* EC_50_ (μM)	*Hs*SR IC_50_ (μM)	HepG2 CC_50_ (μM)	CRL-8155 CC_50_ (μM)	hERG IC_50_ (μM)	Solubility at pH 2 (μM)	Solubility at pH 6.5 (μM)
BKI-1770	0.002	0.5	>10	>80	>80	>30	75	60.2
BKI-1841	0.003	0.7	>10	>80	>80	>30	>100	>100
1770-M1/M1a	<0.122	ND	0.1	1.5	1.4	ND	<1.4	<1
1770-M5	0.018	2.1	5.4	>80	>80	ND	89	>100

a BKI, bumped kinase inhibitor; *Cp*CDPK1, C. parvum calcium dependent protein kinase 1; IC_50_, 50% inhibitory concentration; EC_50_, 50% effective concentration; *Hs*SRC, human tyrosin kinase SRC; CC_50_, 50% cytotoxicity concentration; hERG, human ether-à-go-go-related gene. Data for BKI-1770 has been previously reported (17).

### Pharmacokinetics in mice.

**FIG 2:**
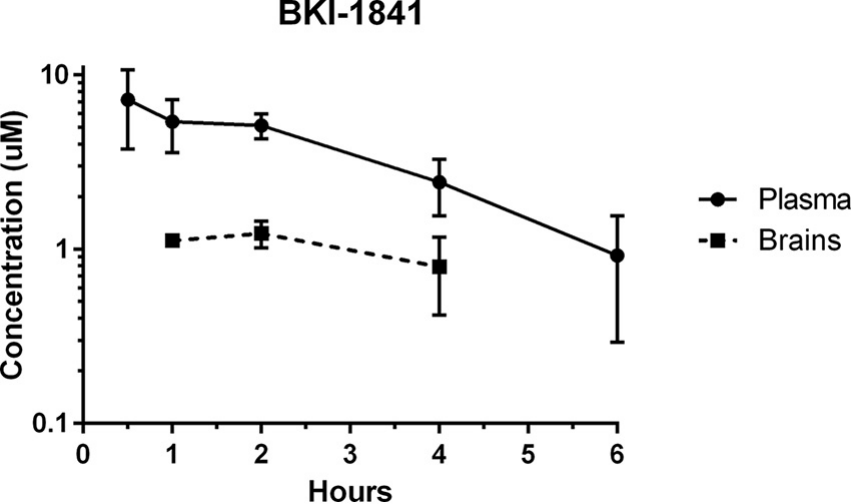
Pharmacokinetics of BKI-1841 in mouse plasma and brain tissue after a single 25 mg/kg oral dose. *n* = 3.

**FIG 3:**
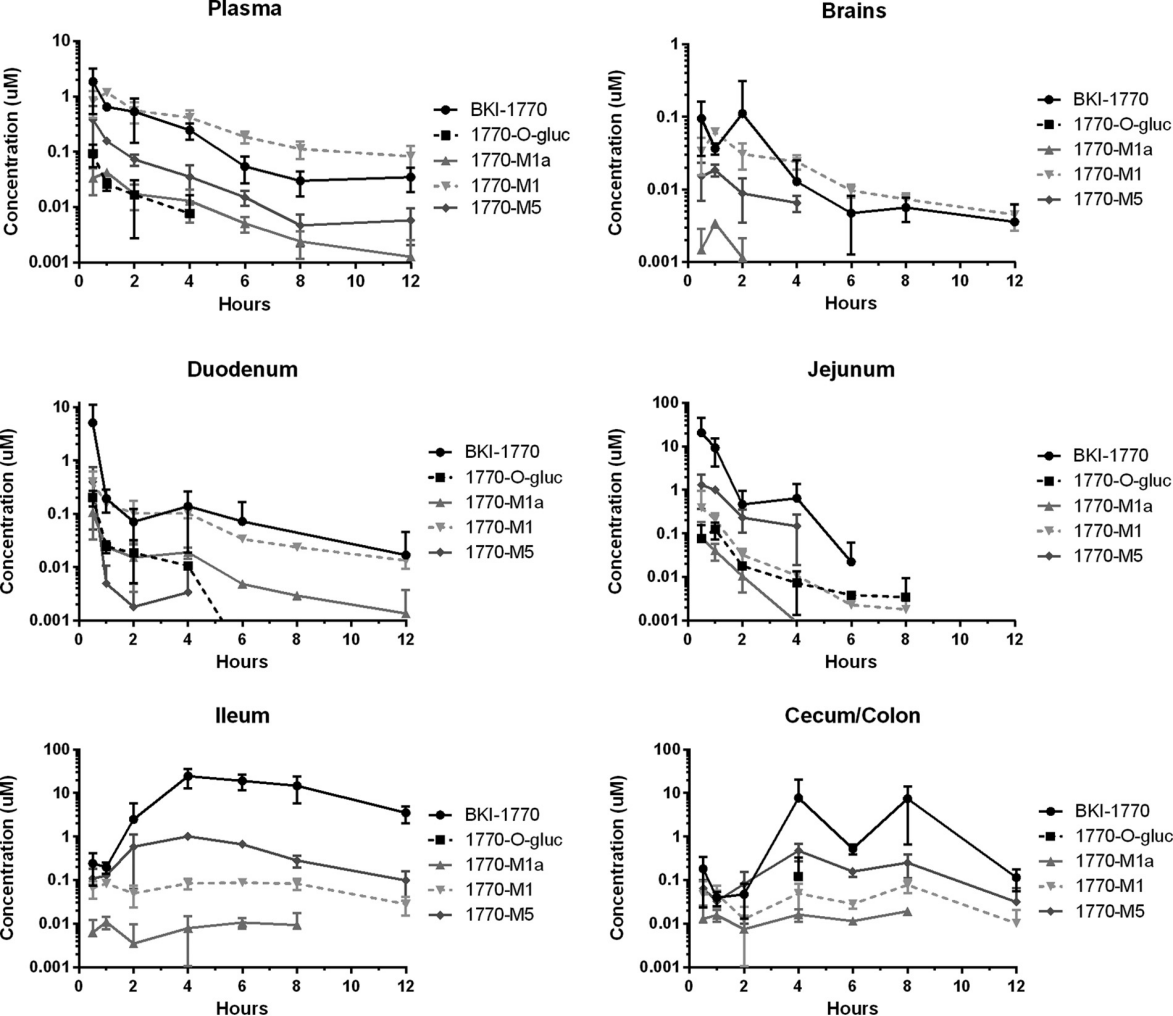
Pharmacokinetics of BKI-1770 and its metabolites in mouse plasma, brain tissue, and gastrointestinal tissues after a single 30 mg/kg oral dose. *n* = 3.

BKI-1770 was previously evaluated for plasma exposure in mice after a single oral dose (17). For evaluation of its suitability for *in vivo* dosing, BKI-1841 was also tested in mice for plasma exposure after a single dose (Fig. 2). BKI-1841 was detectable in plasma at 6 h post dosing at 25 mg/kg and the mice showed no signs of toxicity. A dose of 30 mg/kg of BKI-1770 was used for pharmacokinetic (PK) evaluation of mouse plasma, GI tissues, and brain concentrations of parent compound and metabolites (Fig. 3). A dose of 25 mg/kg BKI-1841 was used to evaluate brain penetration over 4 h using the same method (Fig. 2). Comparison of brain tissue to plasma concentration using area-under-the-curve were determined to be 27% brain penetration for BKI-1841 and 10% brain penetration for BKI-1770.

LC/MS-MS scans of mouse plasma after dosing BKI-1841 failed to detect peaks corresponding to the mass-to charge ratio (*m/z*) of the expected dehydrogenated metabolite of BKI-1841, so this compound was not synthesized for further analysis. However, *m/z* peaks were observed in mouse plasma correlating with some expected metabolites of BKI-1770. These included the products of glucuronylation (1770-O-glucuronide), the major metabolite from all species in hepatocytes; the product of dehydrogenation and subsequent hemiaminal formation 1770-M1 and its further dehydrated product (1770-M1a); and the product of O-dealkylation (1770-M5) (Fig. 1). These compounds were synthesized for evaluation *in vitro* (Table 1) and quantitation of *in vivo* samples. For quantification purposes, 1770-M1/M1a stock was considered 50:50 mix of the hemiaminal 1770-M1 and its dehydrated product 1770-M1a due to the fact that the two products could not be separated during synthesis (Fig. 1). Potentially toxic liabilities were observed for both 1770-M1/M1a and 1770-M5 in their activity against *Hs*SRC (a proxy for off-target, host protein kinase liabilities) and 1770-M1/M1a was potently cytotoxic to mammalian cell lines *in vitro* (Table 1).

### Efficacy in infected mice.

**FIG 4:**
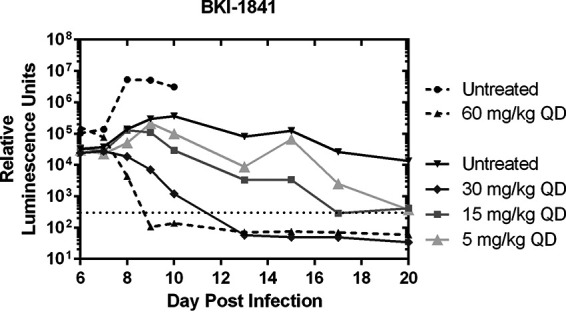
Efficacy of BKI-1841 in interferon-γ knockout mice infected with nanoluciferase expressing C. parvum. Treatments were separated into two separate experiments for BKI-1841. Untreated control groups for each experiment are grouped with their associated treatment in the figure keys. Dotted line denotes the limit of detection. Treatments were dosed on days 6 through 10 postinfection. Significant differences were observed between the control and 60 mg/kg and 30 mg/kg groups (*P* < 0.05) but no significant difference was observed for the 15 mg/kg or 5 mg/kg dose. *n* = 3. QD = once daily.

BKI-1770 was previously shown to be efficacious against Nluc expressing C. parvum in IFN-γ KO mice at a minimum dose of 30 mg/kg twice daily (BID) for 5 days (17). BKI-1841 was also tested at doses of 60, 30, 15, and 5 mg/kg once daily (QD) for 5 days, resulting in a minimum efficacious dose of 30 mg/kg that cleared parasite excretion to background levels by day 13 postinfection (Fig. 4).

### Cardiovascular testing in rats and dogs.

Cardiotoxicity was assessed for both BKI-1770 (21) and BKI-1841 by dosing anesthetized rats with escalating, 30-minute IV infusions of 3, 10, and 30 mg/kg. Rats were observed for changes to mean arterial pressure (MAP), heart rate (HR), and left ventricular contractility (dT/dP@50) (Table S1). No significant effects, considered to be >15% compared to vehicle only controls, were seen for either compound. Additional cardiovascular evaluation was performed on anesthetized dogs using BKI-1770 (21) administered at a rate of 0.02 mL/kg/min. Dogs were observed for 150 min for changes to MAP, HR, dT/dP@50, systemic vascular resistance (SVR), cardiac output (CO), QT intervals (QTcV), QRS intervals, and PR intervals. No appreciable effects were seen in dosed dogs for any of these parameters compared to control animals (Table S2). Thus, neither compound had cardiovascular liabilities in the rat model, and BKI-1770 had no signs of cardiovascular toxicity in the dog model.

### Efficacy in neonatal calves and piglets.

**FIG 5:**
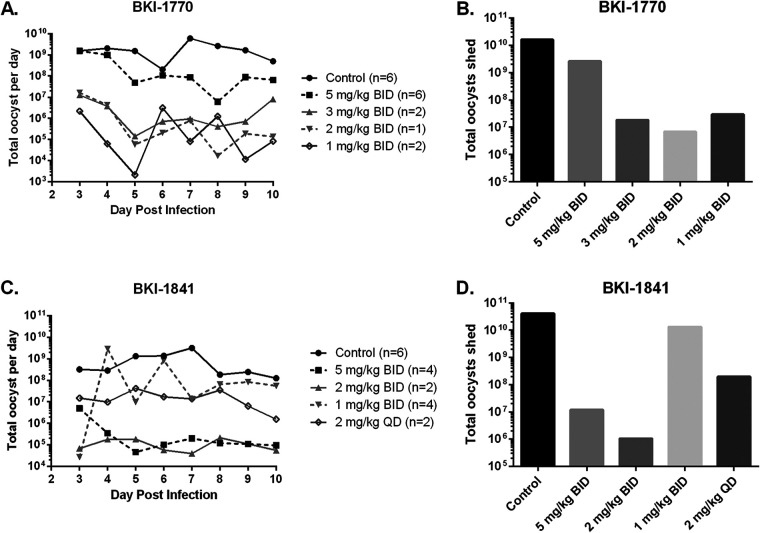
Efficacy of BKI-1770 and BKI-1841 in neonatal calves infected with C. parvum. Total oocyst excretion determined by RT-PCR concentration and total by fecal weight shed per day. (A) Oocyst excretion per day after treatment with BKI-1770. (B) Cumulative total oocyst excretion for days 3 through 10 after treatment with BKI-1770. (C) Oocyst excretion per day after treatment with BKI-1841. (D) Cumulative total oocyst excretion for days 3 through 10 after treatment with BKI-1841. BKI-1770 treatments were administered on days 2 through 6 postinfection. BKI-1841 BID treatments were administered on days 2 through 4 and QD treatments were administered on days 2 through 5. For BKI-1770, significant differences in total oocyst excretion per day were observed for all dosed groups compared to controls (*P* < 0.05). For BKI-1841, significant differences in total oocyst excretion per day were observed for the 5 mg/kg and both 2 mg/kg groups (*P* < 0.05) but no significant difference was observed for the 1 mg/kg group compared to controls. QD = once daily, BID = twice daily.

BKI-1770 and BKI-1841 were both tested for efficacy against C. parvum in infected neonatal calves. BKI-1770 was dosed at 5 mg/kg BID (*n* = 5) for 5 days (Fig. 5, Fig. S3). Total oocyst excretion was significantly lower in treated calves at 2.58E9 total oocysts excreted compared to 1.62E10 for control calves between days 3 and 10 postinfection. Plasma concentrations of BKI-1770 reached an observed maximum concentration (Cmax) level of 10.3 ± 5.4 μM at 4 h post dose 5 (Fig. S3). There were also improvements in the treated calves in both fecal volume and consistency, urine output, weight gain and daily clinical scores compared to control calves (Fig. S3). A dose response experiment was performed with BKI-1770 using doses at 5, 3, 2, and 1 mg/kg BID for 5 days (Fig. 5, Fig. S3). In these experiments, hyperflexion of the limbs, seen as dropped pasterns, were observed in two of the 5 mg/kg calves. One calf treated with 5 mg/kg also showed some bloody diarrhea. In addition to this, the 5 mg/kg dose was less effective than other doses in this experiment. Fecal volume, fecal consistency, and clinical scores were improved for all treatment groups compared to controls, but these improvements were delayed for the 1 mg/kg group. Plasma concentrations were reduced for the 3 mg/kg calves to an observed Cmax of 3.4 ± 0.06 μM at 4 h post dose 1 (Fig. S3), indicating a potentially safe and efficacious dose at 3 mg/kg BID for 5 days.

BKI-1841 was dosed at 5, 2, and 1 mg/kg BID for 3 days and 2 mg/kg QD for 4 days (Fig. 5, Fig. S4). Total oocyst excretion showed a 2 to 4 log reduction for the 5 mg/kg BID for 3 days and 2 mg/kg BID given for 3 or 4 days, but little reduction with the 1 mg/kg BID for 3 days. Clinical scores and weight change were also improved over controls with all doses except 1 mg/kg for 3 days. Urine output was consistent for all groups, including controls, and fecal volume and consistency were improved for all groups over controls (Fig. S4). However, all calves dosed with BKI-1841 showed signs of hyperflexion of the limbs seen as dropped pasterns in their stance that developed shortly after the onset of dosing (Fig. S5). This occurred to some degree with all dosing regimens, developing with the fewest doses at 5 mg/kg BID. With the lowest dose of 1 mg/kg BID, the onset of dropped pasterns began after the third dose and continued until self-resolving 2 days after dosing concluded. All signs of dropped pasterns were gone by the end of the experiment on day 10 postinfection. In addition to dropped pasterns, the 5 mg/kg BID treated group began to show signs of ataxia during the 3 days of dosing. Plasma concentration of BKI-1841 appeared to be accumulating throughout the dosing period (Fig. S4), but decreasing the dose to 1 mg/kg BID still resulted in dropped pasterns despite only reaching predose plasma concentrations of 1.3 ± 0.1 μM by the last day of treatment (Fig. S4).

**FIG 6:**
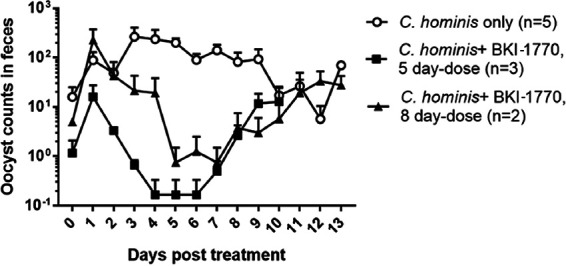
Efficacy of BKI-1770 in neonatal piglets infected with C. hominis. Piglets were inoculated orally with C. hominis oocysts 2 days after birth and treatment with BKI-1770 at 5 mg/kg BID began 3 days postchallenge (day 0 post treatment). Rectal swabs were processed for oocyst count and DNA measurement. The plot represents mean ± SEM. Wilcoxon matched-pairs signed rank test was conducted using GraphPad Prism 7.03. Significant differences were observed between controls and the 5 day-dose (*P* = 0.001) and the 8 day-dose (*P* = 0.01). The 5 day doses were given on days 0–4, the 8 day doses were given on days 0–7.

BKI-1770 was also tested for efficacy against C. hominis in infected neonatal gnotobiotic piglets with a dose of 5 mg/kg BID for 5 or 8 days (Fig. 6, Fig. S6). Oocyst counts in feces were significantly reduced in both the 5-day treatment (*P* = 0.001) and 8-day treatment (*P* = 0.0168) compared to controls. Diarrhea scores were also significantly reduced for both treatment groups while plasma levels remained low at all collected time points (Fig. S6). Plasma levels of the piglets reached an observed Cmax of 2.1 ± 1.4 μM at 2 h post dose 5 and 0.9 ± 0.4 μM at 2 h post dose 8 (Fig. S6), substantially lower than concentrations reached in calves at the same 5 mg/kg BID dose. Treated piglets showed no visible signs of toxicity.

### Multidose rat toxicity testing.

Due to the promising efficacy of BKI-1770 in both calves and piglets, the compound was tested in a 14-day multidose rat toxicity study. Male and female rats were administered BKI-1770 orally with 375, 75, or 15 mg/kg QD. Rats administered 15 mg/kg showed no signs of toxicity. Rats administered 75 mg/kg showed some signs of bone toxicity, including thickened epiphyseal plates and increased spongiosa. This group also showed signs of necrosis and/or inflammation in the liver, common bile duct, pancreas, spleen, lymph nodes, stomach, heart, and kidney, atrophy in the thymus, thyroid, and vagina, and hypertrophy in the adrenal gland. Rats dosed with 375 mg/kg were euthanized after 7 doses due to extreme signs of toxicity. The plasma concentrations ranged from 11.8 μM in the 375 mg/kg group to 2.4 μM in the 15 mg/kg group.

### Multidose calf toxicity testing.

BKI-1770 was also tested in a multidose calf toxicity study. Male and female calves were dosed orally with 7.5 or 15 mg/kg BID for 5 days. Adverse effects began on day 4, so the 7.5 mg/kg group received 9 doses total and the 15 mg/kg group received 7 doses total before the experiment was terminated. Calves dosed with 7.5 mg/kg began showing locomotion problems on day 3, including swaying and pattering, and difficulty getting up before quickly laying back down again. Calves dosed with 15 mg/kg began showing locomotion problems on days 2 and 3, including pattering, swaying, lack of coordination, ataxia, and severe hyperflexion of the limbs seen as dropped pasterns. Both the 7.5 and 15 mg/kg groups showed changes to the epiphyseal growth plates in the femur and sternum, indicating a disruption to normal bone growth (Fig. S5). No other significant signs of toxicity could be associated with BKI-1770 treatment.

### Multidose mouse toxicity.

**FIG 7:**
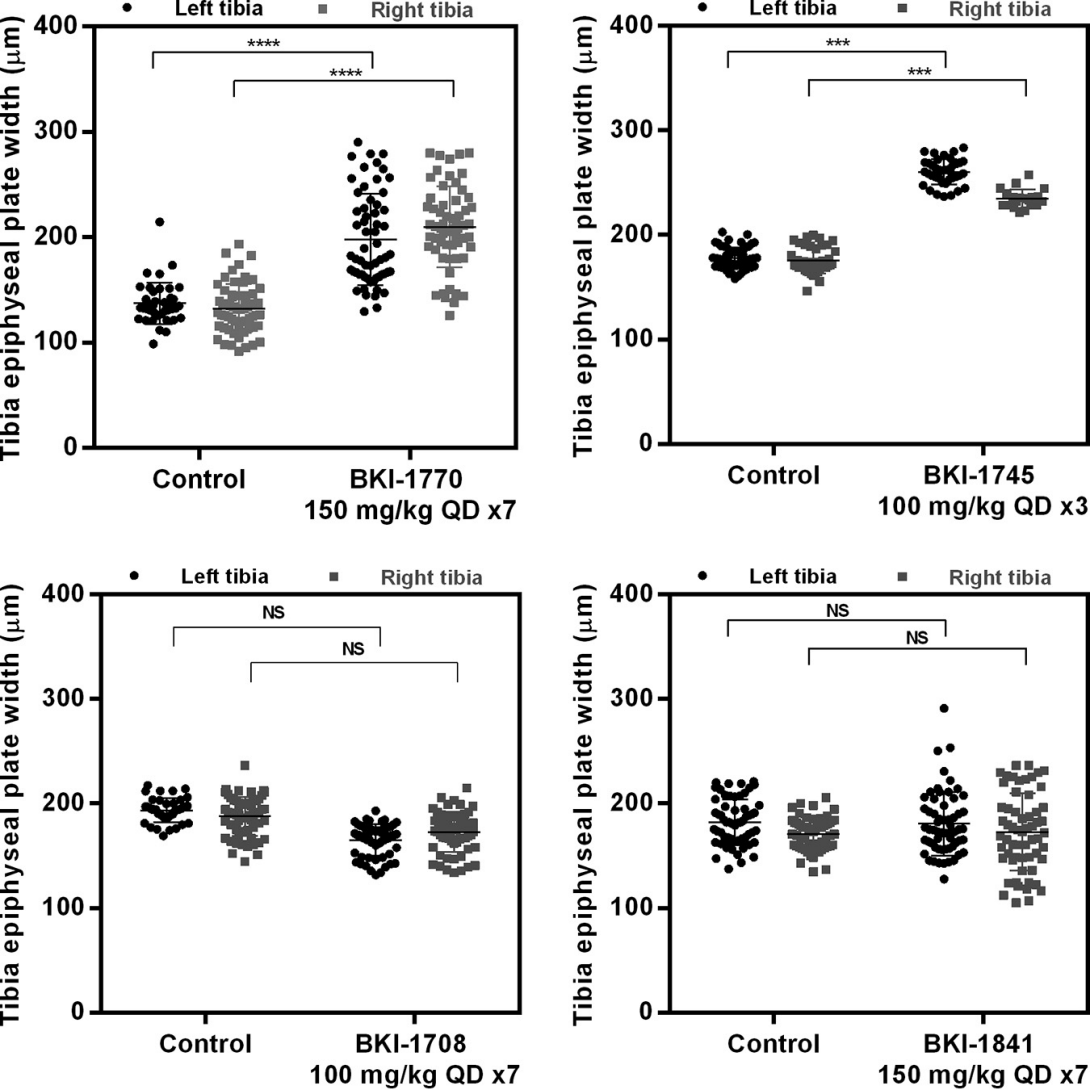
Bone toxicity of BKI-1770, BKI-1745, BKI-1708, and BKI-1841 as observed in changes to the width between the borders of the epiphyseal growth plate of the left and right tibia in 3–4 week old mice. Mice were dosed by oral gavage with either 100 mg/kg (BKI-1745, -1708) or 150 mg/kg (BKI-1770, -1841) QD for 7 days and euthanized 24 h after the final dose. Tibia were removed and processed for staining immediately after euthanasia. Statistical significant differences (ANOVA; *P* < 0.001) were observed for BKI-1770 and BKI-1745. *n* = 3. QD = once daily, NS = not significant.

The recurring presence of toxicity in calves manifesting as dropped pasterns, as well as the observations of bone toxicity in rats, led to the exploration of possible bone toxicity screening in mice to allow for screening prior to dosing in larger animals. To test for effects to the growth plate in developing bones, 3 week old mice were dosed for 7 days QD and euthanized 24 h after the final dose. The right and left tibia were dissected from each mouse and stained with AgNO_3_ to visualize the growth plate and measurements across the widths compared to vehicle-only treated controls. This was performed with four compounds, BKI-1770 at 150 mg/kg, BKI-1841 at 150 mg/kg, BKI-1745 at 100 mg/kg, and BKI-1708 at 100 mg/kg (Fig. 7). BKIs -1708 and -1745 were previously shown to be efficacious in mice (17), so were included as potential future candidates to rule out possible toxicity before being advanced to additional *in vivo* studies. Mice dosed with BKI-1745 showed extreme signs of toxicity by the third dose, with one mouse dying unexpectedly prior to the fourth scheduled dose. The remaining two mice for this treatment were immediately euthanized and their tibias extracted early on day 4. Mice dosed with BKIs -1770, -1708, and -1841 showed no gross signs of toxicity through all seven doses. BKIs -1770 and -1745 both showed significant (*P* < 0.05) increases in the width of the epiphyseal growth plate. Conversely, BKIs -1708 and -1841 did not show significant increases to the width of the epiphyseal plate. Blood chemistry and histology were previously performed on both BKIs -1770 and -1708 with no apparent abnormalities compared to controls animals (17). Blood chemistry and histology on mice dosed with BKI-1841 showed no abnormalities or indications of toxicity compared to control animals (data not shown).

### Mouse locomotor activity box testing.

Since the dropped pasterns in calves self-resolved quickly after the cessation of treatment and there were no affects to the epiphyseal plate in mice treated with BKI-1841, possible neurological causes for the toxicity observed in calves were explored. BKIs –1770, -1841, and -1708 were tested in the mouse locomotor activity box to screen for possible neurological issues (Fig. S7). All three BKIs were dosed at 150 mg/kg QD for 5 days. BKI-1708 treated mice showed no inhibitory effects in locomotor activity, and led to a statistically significant increase in distance traveled from 40 to 60 min and vertical rearing activity from 0 to 20 and 40 to 60 min. BKI-1841 treated mice showed a significant (*P* < 0.05) decrease in distance traveled, stereotypic horizontal activity and vertical rearing activity from 0 to 60 min. BKI-1770 treated mice showed a significant (*P* < 0.05) decrease in distance traveled from 20 to 60 min, increased horizontal activity from 0 to 40 min and decreased vertical activity throughout the study compared to controls. Thus, both BKI-1841 and BKI-1770 showed a depression in movement compared to controls and BKI-1708, suggesting the dropped pastern effect and the ataxia could be CNS issues that can also be detected in rodents.

## DISCUSSION

The BKIs under study here differed in the dose interval required to get efficacy, in that BKI-1841 was efficacious when dosed daily in mice and calves, but BKI-1770 required twice daily dosing in mice and calves. BKI-1841 showed prolonged plasma exposure of >1 μM for more than 6 h after a single 25 mg/kg dose in mice and its most probable metabolite was absent *in vivo*, suggesting some degree of metabolic stability. In contrast, BKI-1770 was rapidly depleted in the plasma to submicromolar concentrations within 1 to 2 h after oral dosing. This may explain why BKI-1841 showed strong efficacy in mice using only QD dosing while BKI-1770 required BID dosing, as it has been previously shown that BKIs work through interference with cell invasion and egress (24–27) and therefore require a sustained presence for strong efficacy against *Cryptosporidium in vivo* (18). BKI-1770 also showed high and or sustained concentrations in each section of the GI tract tissues after a single dose. In addition to sustained exposure, it was also previously shown that BKIs must gain sufficient exposure to tissues in all sections of the GI tract to be efficacious (28). BKI-1770 reached high peak concentrations in both the duodenum and jejunum, which was repeated twice daily throughout the dosing period, offering repeated exposure to concentrations that are four to 15 times the 50% effective concentration for the compound. Additionally, concentrations in the ileum and cecum/colon tissues rose to high and sustained levels through at least 8 h after dosing, with ileum concentrations remaining at >1 μM through 12 h. The main mechanism of metabolism, as indicated by the relative abundance produced in hepatocytes for all tested species (Fig. S2), was glucuronylation of BKI-1770 to 1770-O-glucuronide. This could have also contributed to sustained levels throughout the gut. If the 1770-O-glucuronide is subject to enterohepatic recirculation, it probably is enzymatically hydrolyzed in the gut back to its parent compound, thereby increasing BKI-1770 exposure within the GI tract. This is one possible explanation for why the terminal phase shifts to a different rate within the duodenum and jejunum tissues at around 2 h post dose. It could also explain the double peaks in concentration for the cecum/colon, where the first peak would represent initial drug passing and the second would represent the 1770-O-glucuronide gut microbiome hydrolyzed product after recirculation. The 1770-O-glucuronide being metabolized in the GI tract is also supported by the fact that it appears only at low levels in the duodenum and jejunum with rapid elimination and does not appear in the lower sections of the GI tract at all. Further studies are warranted to discover whether glucuronylation and enterohepatic recirculation could benefit the efficacy of cryptosporidiosis treatment with similar compounds.

The observations of hyperflexion to the limbs seen as dropped pasterns in the calves may be caused by either bone toxicity or neurological toxicity, or a combination of both. Other causes seem less likely since no other signs of toxicity were observed in the calves or in rodents. It is questionable that something as severe as an alteration of the growth plate would resolve quickly after cessation of treatment, as was seen with calves dosed with BKI-1841, suggesting a possible neurological cause. BKI-1770 showed bone toxicities in calves, rats, and mice. Since BKI-1770 and BKI-1841 both readily penetrate the CNS and both exhibited significant effects in the mouse locomotor activity box, neurological toxicity could account for the calf findings of dropped pasterns and ataxia from BKI-1841, which were temporary and associated with dosing, while BKI-1770 may carry the combined effects of neurological toxicity and bone toxicity. This combined bone and neurological toxicity may have led to the dropped pasterns and ataxia that appeared in two of the calves dosed at the 5 mg/kg in the calf efficacy and all of the calves dosed with BKI-1770 7.5 mg/kg and 15 mg/kg BID in the toxicity experiment. It is possible that the BKI-1770 metabolites 1770-M1/M1a, which show potential signs of toxicity *in vitro*, and are present in high concentrations in plasma and brain tissue at time points after 2 to 4 h post dose in mice, could be causing toxicity directly in calves, if these metabolites accumulate.

Calf efficacy experiments indicated that BKI-1770 given at 5 mg/kg BID was reaching the threshold of toxicity in some animals, since two of the six calves dosed began showing dropped pasterns. The experiment with the BKI-1841 5 mg/kg BID treated calf that developed bloody diarrhea began with substantially lower levels of cryptosporidium infection (Fig. 5) than the previous 5 mg/kg BID (*n* = 5) experiment, suggesting that the bloody diarrhea observed in that calf was either due to another pathogen infection or an infrequent effect of BKI-1770, but probably not associated with C. parvum infection. The lower dose of BKI-1770 3 mg/kg BID outperformed 5 mg/kg BID in reducing oocyst excretion, and improved fecal consistency, fecal volume, urine output, and clinical scores, though 5 mg/kg still showed significant improvements over controls. Since toxicity only appeared in two out of six calves dosed at 5 mg/kg BID, and not at lower doses, and piglets demonstrated lower plasma concentrations at the same dose with no observable signs of toxicity, BKI-1770 may be safer at lower plasma concentrations. Many drug developers assess a compound’s margin of safety using the safety index, which is calculated by the ratio of the plasma exposure obtained at lowest “safe” dose divided by the exposure obtained from the lowest efficacious dose. A safety index of >10 is desirable for an infectious disease therapeutic, and safety is particularly important for a therapeutic targeting 6 to 18 month-old malnourished children. Unfortunately, given the relatively low plasma concentrations of rats displaying bone and organ toxicity for BKI-1770, and the relatively high plasma concentrations of BKI-1770 in calves dosed with the minimal efficacious dose, 3 mg/kg BID, it is very unlikely that an acceptable >10 safety index would be found. Similarly, calves displaying dropped pasterns for BKI-1841 occurred even at the lowest dose tested (1 mg/kg BID), even when losing efficacy for cryptosporidiosis, suggesting a safety index of <1. Thus, it is unlikely either of these compounds can demonstrate a large enough safety index to remain preclinical candidates for cryptosporidiosis.

Although many *in vitro* screens can warn of potential *in vivo* toxicities, *in vivo* testing often reveals signs of toxicity that could not otherwise be predicted. Escalating doses *in vivo* toxicity studies serves to collect this important safety data, as was done with BKI-1770. This was the case when BKI-1770 was dosed in cattle and rats, leading to the observations of thickening epiphyseal plates. Once this was observed, along with the hyperflexion of the limbs (dropped pasterns) with BKI-1841, compound testing was halted in large animals in the hopes that these toxicities could first be screened out in mice. The results of epiphyseal plate thickening for BKI-1770 and CNS toxicity effects for both BKIs-1770 and -1841 in mice showed agreement with toxicities observed in rats and calves, allowing for the earlier identification of these problems and the possibility of determining toxicities without the need for larger animals, which require significantly more resources than small rodents. Since BKI-1708 did not show changes to the epiphyseal plates in mice and showed no inhibition of activity in the mouse locomotor activity box, this compound continues to be a viable candidate for further evaluation as a potential treatment for cryptosporidiosis in both animals and humans and can be moved into larger animal models with an increased confidence that these liabilities are less likely to occur, having already been screened out in the mouse models.

## MATERIALS AND METHODS

### Previously described methods.

Methods for synthesis of BKIs (17, 23), efficacy against Nluc expressing C. parvum in infected adult IFN-γ KO mice (6, 9), *in vivo* mouse GI tract tissue exposure by LC/MS-MS analysis (18, 28), PK analysis of mouse plasma and brain tissue BKI concentrations by LC/MS-MS analysis (29), *in vivo* rat and dog cardiotoxicity screen (30), efficacy against IOWA-II strain C. parvum in infected neonatal calves (13), efficacy against C. hominis in infected piglets (12), *in vitro* inhibition of C. parvum CDPK1 (*Cp*CDPK1) and human tyrosine kinase SRC (*Hs*SRC) activity (31), *in vitro* inhibition of hERG (32), Nluc expressing C. parvum *in vitro* growth inhibition in infected HCT-8 cells (6), *in vitro* cytotoxicity in CRL-8155 and HepG2 cells (29), and mouse locomotor activity box (33, 34) have all been previously described. Analytes were measured by LC/MS-MS on an Acquity ultra performance liquid chromatography (UPLC) system in tandem with a Xevo TQ-S mass spectrometer (Waters, Milford, MA, USA).

### Compound synthesis: 5-Amino-3-(6’-ethoxynaphthalen-2’-yl)-1-(4’’-hydroxy-2’’-methylbutan-2’’-yl)-1H-pyrazole-4-carboxamide *O*-glucuronide, ammonium salt (1770-O-glucuronide).

The glucuronylation reaction was performed using the E. coli glucuronylsynthase enzyme and α-d-glucuronyl fluoride according to the literature (35–37). BKI-1770 (5.0 mg, 13 μmol, 0.35 mM final concentration) was dissolved in *tert*-butanol (3.73 mL, 10% vol/vol of total volume) and sodium phosphate buffer (31.55 mL, 50 mM, pH 7.5). The E. coli E504G glucuronylsynthase (677 μL, 11 mg/mL, final concentration 0.2 mg/mL) and α-d-glucuronyl fluoride (60 mg, 0.28 mmol, 21 equivalents) dissolved in sodium phosphate buffer (1.30 mL, 50 mM, pH 7.5) were added and the reaction mixture was incubated without agitation at 37°C for 2 days. The reaction was then subjected to solid-phase extraction (SPE). An Oasis Weak Anion Exchange (WAX) SPE cartridge (500 mg, 6 mL) was preconditioned with methanol (5 mL) and milliQ water (15 mL). The reaction mixture was loaded onto the cartridge and eluted under a positive pressure of nitrogen at a flow rate of approximately 2 mL/min with the following solutions: formic acid in water (2% vol/vol, 25 mL), water (25 mL), methanol (50 mL) and saturated aqueous ammonia solution in methanol (5% vol/vol, 25 mL). The methanolic ammonia fraction was concentrated *in vacuo* to yield 1770-O-glucuronide, ammonium salt as a clear oil (0.6 mg, 8% yield). The H1''' signal showed a correlation to C4'' in the HMBC spectrum confirming the formation of the *O*-glucuronide. The purity of final product was confirmed by HPLC-UV to be greater than 95%. Additional data are included in the Supplemental Material (Fig. S1).

### BKI-1770 metabolite compounds.

Data for the synthesis of 1770-M1/M1a and 1770-M5 (Fig. 1) are included in Supplemental Material (Fig. S1).

### Multidose toxicity in rats and calves.

Rat toxicity testing was performed by oral dosing of BKI-1770 to male and female rats in 10% ethanol/40% kolliphor/50% water once daily for 14 days. A control group of male and female rats were administered vehicle only for controls. Male and female calves were dosed orally twice daily with BKI-1770 in PEG200 or vehicle only for 5 days. Observations of feeding and general health were made twice daily. Clinical assessments were made at 1 h after each treatment. Blood was collected for hematology and blood chemistry before the first dose and prior to euthanasia. Necropsy and histology of organs (Liver, heart, lung, kidney, spleen, thymus, brain, spinal cord, nerves of the front and back legs, including muscle, bone marrow femur, stomach, small and large intestine, including lymph nodes, and femur and sternum, including epiphyseal plates, distal hind limb, and thyroid gland) were performed after euthanasia.

### Multidose toxicity in mice.

Mouse toxicity testing and evaluation of the tibial epiphyseal plate have been previously described (18, 38) and were performed with some modifications. Briefly, 3 week old BALB/c mice (*n* = 3) were dosed orally once daily for 7 days. Mice were euthanized along with vehicle treated controls by cardiac puncture for collection of blood for analysis. Necropsy, obtaining blood for serum chemistries, histology of organs (brain, heart, lungs, spleen, liver, stomach, small intestine, large intestine and kidney), and removal of tibias were performed within 24 h of the final dose. Examination of organ histology included routine H&E and Oil-Red O (lipid)-stained organs from the treated mice and a set of untreated matched controls. Pathological examination was performed by a board certified pathologist who was blinded to the treatment and control group identities. Tibias were removed and immediately placed in dH_2_O for a minimum of 1 h. Using a fresh number 11 scalpel blade, a quick sagittal cut was made across the femoral condyle opposite the fibula, and a second cut was made across the second femoral condyle separating the fibula and tibia. The tibias were then immersed in acetone for 1 h to dehydrate. Dehydrated tibias were washed in dH_2_O for 2 min, then soaked in 1.5% AgNO_3_ for 1 min in a container protected from light. Tibias were then rinsed in dH_2_O for 2 min and exposed to strong light for 10 sec. Stained tibias were stored in 70% ethanol for >10 min before examining the growth plates at ×40 magnification using an Amscope stereo microscope with a mounted camera. Photos were taken using the Amscope image capture software. A calibration slide was used to establish a μm to pixel ratio of 1:1.184. Using the Measurement Tool, 10 measurements were taken across each epiphyseal plate by orienting the cursor at right angles from the growth plate borders by two different researchers (one set unblinded; one set blinded) and average width was assigned to each tibia from each animal.

### Animal ethics.

All animal experiments conducted at the University of WA, USA, the University of Arizona, and USA, Tufts University, USA, were approved their Institutional Animal Care and Use Committees. Animal experiments performed at AbbVie were reviewed and approved by AbbVie's Institutional Animal Care and Use Committee and conducted in an AAALAC accredited program where veterinary care and oversight was provided to ensure appropriate animal care. All animals used in these experiments were handled in strict accordance with practices made to minimize suffering.
