# Supplementary material for: Comparison of Toxicities among Different Bumped Kinase Inhibitor Analogs for Treatment of Cryptosporidiosis
Source: Antimicrob Agents Chemother. 2023 Mar 15;67(4):e01425-22. doi: 10.1128/aac.01425-22 (PMC10112232; doi:10.1128/aac.01425-22)
Supplement: Supplemental file 1 — Supplemental material. Download aac.01425-22-s0001.pdf, PDF file, 1.9 MB [file aac.01425-22-s0001.pdf]

Supplemental Materials for Comparison of toxicities among different bumped kinase inhibitor analogs for treatment of cryptosporidiosis

Matthew A. Hulverson[a], Ryan Choi[a], Deborah A. Schaefer[b], Dana P. Betzer[b], Molly C. McCloskey[a], Grant R. Whitman[a], Wenlin Huang[c], Sangun Lee[d], Andy Pranata[e], Malcolm D. McLeod[e], Kennan Marsh[f], Dale J. Kempf[f,g], Bruce E. LeRoy[f], Mark T. Zafiratos[f], Aimee L. Bielinski[f], Robert C. Hackman[h,I,j], Kayode K. Ojo[a], Samuel L.M. Arnold[a], Lynn K. Barrett[a], Saul Tzipori[d], Michael W. Riggs[b], Erkang Fan[c], and Wesley C. Van Voorhis[a, #].

[a] Department of Medicine, Division of Allergy and Infectious Disease, Center for Emerging and Reemerging Infectious Disease (CERID), University of Washington, Seattle, WA 98109, USA

[b] School of Animal and Comparative Biomedical Sciences, College of Agriculture and Life Sciences, University of Arizona, Tucson, AZ 85721, USA

[c] Department of Biochemistry, University of Washington, Seattle, WA 98195, USA

[d] Department of Infectious Disease and Global Health, Cummings School of Veterinary Medicine at Tufts University, North Grafton, MA 01536, USA

[e] Research School of Chemistry, Australian National University, Acton, ACT 2601, Australia

[f] Research and Development, AbbVie, Inc, North Chicago, IL 60064, USA

[g] Former employee of Research and Development, AbbVie, Inc, North Chicago, IL 60064, USA

[h] Fred Hutchinson Cancer Research Center, Seattle, WA 98109, USA

[i] Department of Pathology, University of Washington, Seattle, WA 98195, USA

[j] Department of Laboratory Medicine, University of Washington, Seattle, WA 98195, USA

[#] Corresponding author. E-mail address: wesley@uw.edu (W.C. Van Voorhis).

Supplemental Figure 1. Compound synthesis of (A) 5-amino-3-(6'-ethoxynaphthalen-2'-yl)-1-(4''-hydroxy-2''-methylbutan-2''-yl)-1H-pyrazole-4-carboxamide *O*-glucuronide, ammonium salt (1770 *O*-glucuronide), (B) product of BKI-1770 dehydrogenation to 1770-M1 and subsequent dehydration to 1770-M1a, and (C) product of BKI-1770 O-dealkylation to 1770-M5.

A.

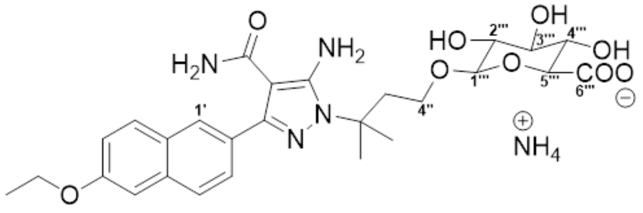

^{1}H NMR (400 MHz, CD_3OD): 7.94 (s, 1H), 7.87-7.82 (m, 2H), 7.56 (d, *J* = 8.4 Hz, 1H), 7.28 (s, 1H), 7.18 (m, 1H), 4.26 (d, *J* = 7.8 Hz, 1H, C1'''-H), 4.18 (q, *J* = 7.0 Hz, 2H, OC<u>H$_2$</u>CH_3), 4.04 (m, 1H, C4''-H_A), 3.60-3.54 (m, 1H, C4''-H_B), 3.57 (d, *J* = 9.4 Hz, 1H, C5'''-H), 3.44 (t, *J* = 9.2 Hz, 1H, C4'''-H), 3.37 (t, *J* = 8.7 Hz, 1H, C3'''-H), 3.18 (t, *J* = 8.3 Hz, 1H, C2'''-H), 2.42-2.27 (m, 2H, C3''-H_2), 1.72 (s, 6H, 2 x CH_3), 1.47 (t, *J* = 6.9 Hz, 3H, OCH_2C<u>H$_3$</u>), NH and OH not observed; ^{13}C NMR (150 MHz, CD_3OD): 176.3 (C6''', assigned by HMBC cross peak analysis), 169.6, 159.1 (C7'), 152.7, 149.7, 136.2, 130.8, 130.1, 129.9, 129.5, 128.4, 128.4, 120.8, 107.5, 104.5 (C1'''), 96.9, 77.9 (C3'''), 76.2 (C5''', assigned by HSQC cross peak analysis), 75.0 (C2'''), 73.7 (C4'''), 67.4 (C4''), 64.6 (O<u>C</u>H_2CH_3), 62.1, 41.0 (C3''), 28.0 (2C), 15.1 (OCH_2<u>C</u>H_3); LRMS (-ESI): 557 (100%, $[C_{27}H_{33}N_4O_9]^-$, $[M-H]^-$); HRMS (-ESI): found 557.2246, $[C_{27}H_{33}N_4O_9]^-$ requires 557.2242.

SAMPLE INFORMATION

Sample Name:	AP115B97-all-final	Acquired By:	System
Sample Type:	Unknown	Sample Set Name:	AndyWESGLUCtrials_Nov2018
Vial:	70	Acq. Method Set:	WesGluc1
Injection #:	1	Processing Method	WesGLUCs
Injection Volume:	5.00 ul	Channel Name:	254.0nm
Run Time:	15.0 Minutes	Proc. Chnl. Descr.:	PDA 254.0 nm
Date Acquired:	27/11/2018 9:04:32 PM EST		
Date Processed:	28/11/2018 11:18:10 AM EST		

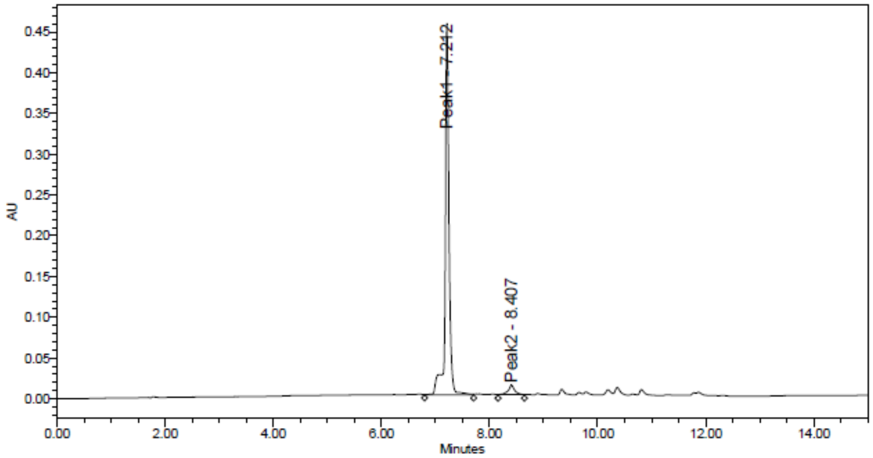

	Peak Name	RT	Area	% Area	Height
1	Peak1	7.212	2244860	95.15	455909
2	Peak2	8.407	114384	4.85	12284

B.

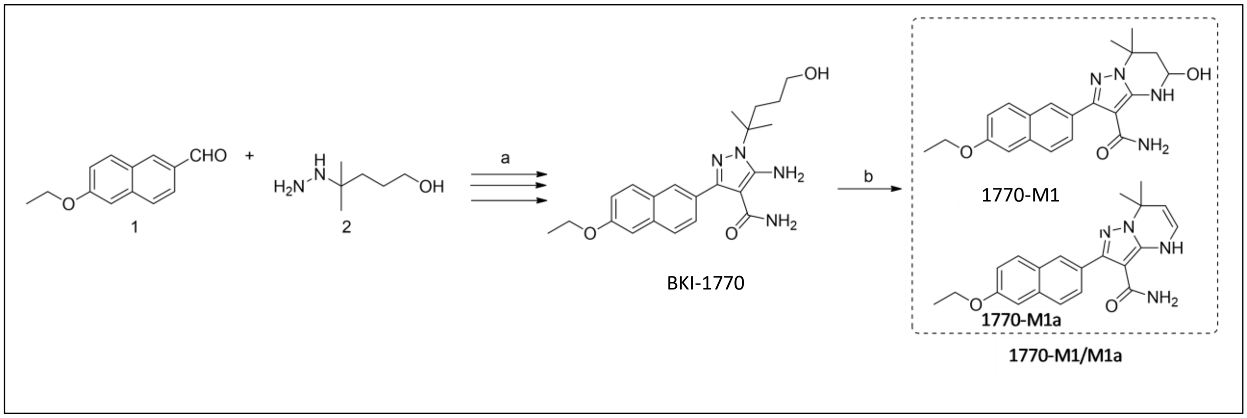

Scheme 1: (a) Reported procedure (1); (b) Dess-Martin reagent, $NaHCO_3$, CH_2Cl_2, 1h.

Compound 1770 (3.8 mg, 0.01 mmol) and sodium bicarbonate (5 mg, 0.06 mmol) were suspended in DCM (4 mL) at 0 °C. Dess Martin reagent (12.7 mg, 0.3 mmol) was added in portions and stirred at 0 °C for 1 hour. The reaction was diluted with DCM and washed by water. The organic layer was dried over Na_2SO_4, filtered, and concentrated in vacuo. The crude product was purified via flash chromatography over silica, eluting with 60% EA in hexane to get compound 1770-M1/M1a (0.83 mg, 21.8%). The purified product exists as 1770-M1 in organic solvent as shown by NMR. In reverse phase LCMS analysis, water adduct 1770-M1a is also present.

NMR of 1770-M1: ^{1}H NMR (500 MHz, $CDCl_3$) δ 7.98 (s, 1H), 7.94 (s, 1H), 7.79 (dd, *J* = 14.2, 8.7 Hz, 2H), 7.62 (d, *J* = 8.3 Hz, 1H), 7.19 (d, *J* = 8.9 Hz, 1H), 7.15 (s, 1H), 6.20 (dd, *J* = 7.8, 4.6 Hz, 1H), 4.64 (d, *J* = 8.0 Hz, 1H), 4.17 (q, *J* = 6.9 Hz, 2H), 1.71 (s, 6H), 1.50 (t, *J* = 6.9 Hz, 3H).

1770-M1 MS (ESI) $(M+H)^+$= 381.148>114.884, 381.148>262.049, 381.148>308.024.

1770-M1a MS (ESI) $(M+H)^+$= 363.01>247.074, 363.074>304.051.

C.

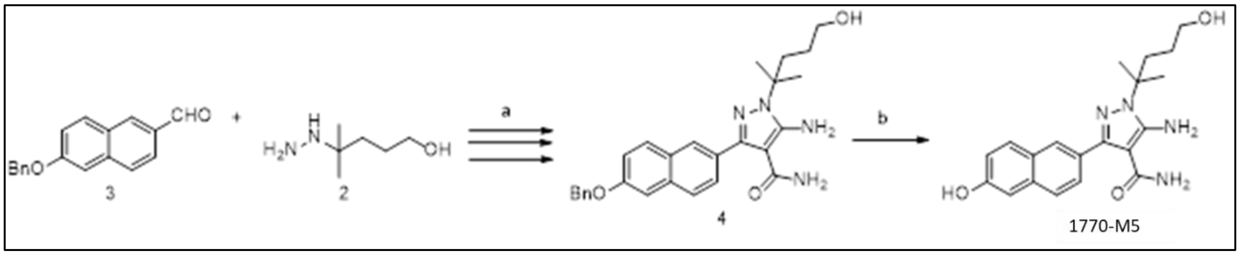

Scheme 2: (a) Reported procedure (1); (b) Pd/C, H_2, CH_3OH, r.t. overnight.

To compound 4 (4.6 mg, 0.01 mmol) dissolved in CH_3OH 5 mL was added Pd/C (10 mg), the flask was then purged with N_2 and filled with H_2. The mixture was reacted at r.t. overnight. The organic layer after filtration was dried over Na_2SO_4, concentrated and purified by flash column chromatography over silica, eluting with 4% CH_3OH in CH_2Cl_2 to get compound 1770-M5 (2.6 mg, 70.7%).

1770-M5 MS (ESI) $(M+H)^+$= 355.074>252.027, 355.074>269.084, 355.074>269.086.

Supplemental Figure 2. Relative abundance of: (A) BKI-1770 metabolites in hepatocytes of 5 species. M1 represents BKI-1770 metabolite created by dehydrogenation, hemiaminal formation, and dehydration of the hemiaminal, M2 represents metabolite formed by oxidation, M3 represents metabolite formed by glucuronylation, M4 represents metabolite formed by hydroxylation, M5 represents metabolite formed by O-dealkylation, and M6 represents metabolite formed by glucuronylation of M5. (B) BKI-1841 in hepatic S9 fractions of 5 species. M1-2 peak represents metabolite of BKI-1841 created by dehydrogenation. See Figure 1 of manuscript for structures.

A.

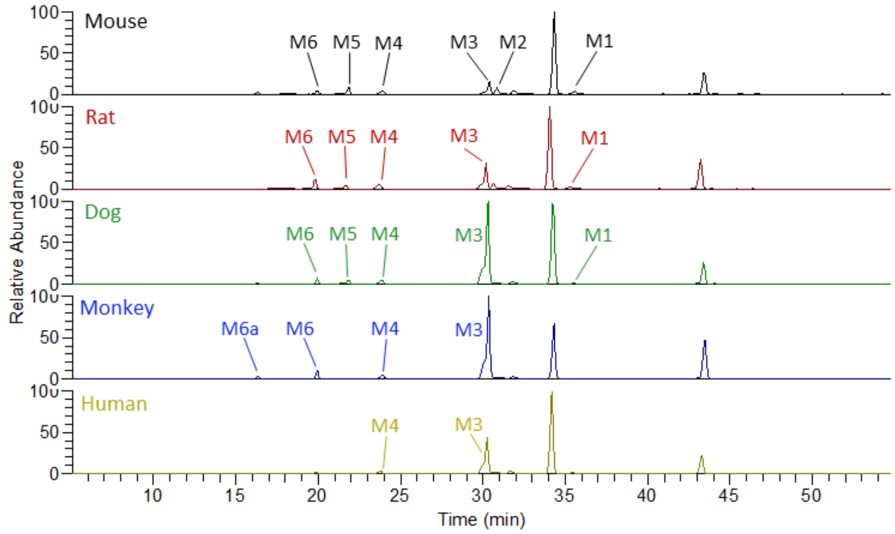

B.

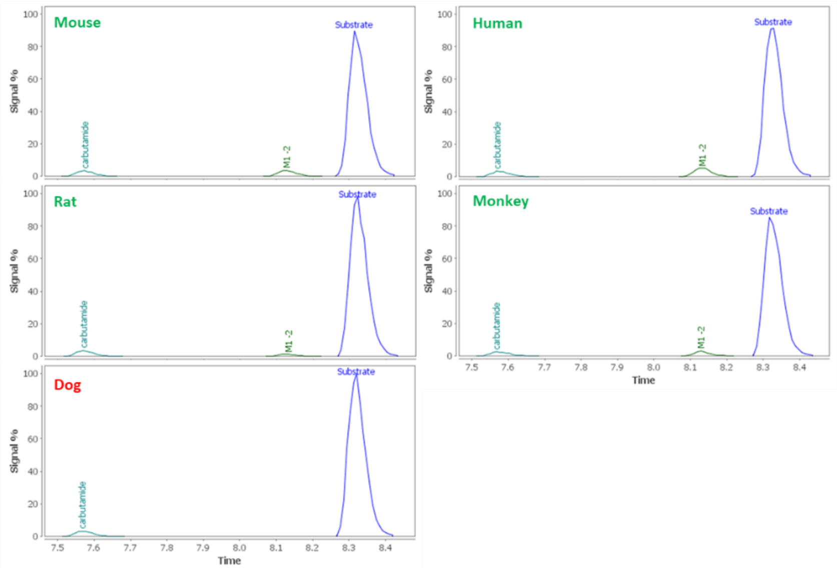

Supplemental Figure 3. (A) Daily and total average fecal consistency, fecal volume, clinical scores and total average urine output and percent weight gain of calves infected with *C. parvum* and treated with BKI-1770 at 5 mg/kg (n=6), 3 mg/kg (n=2), 2 mg/kg (n=1), and 1 mg/kg (n=2) BID for 5 days. (B) Plasma concentrations of BKI-1770 during dosing. Calves dosed with 5 mg/kg numbered as 659, 660, 931, 933, and 179. Calves used in dose response at 5, 3, 2, and 1 mg/kg numbered as 415, 419, 414, 38, 40, and 39.

A.

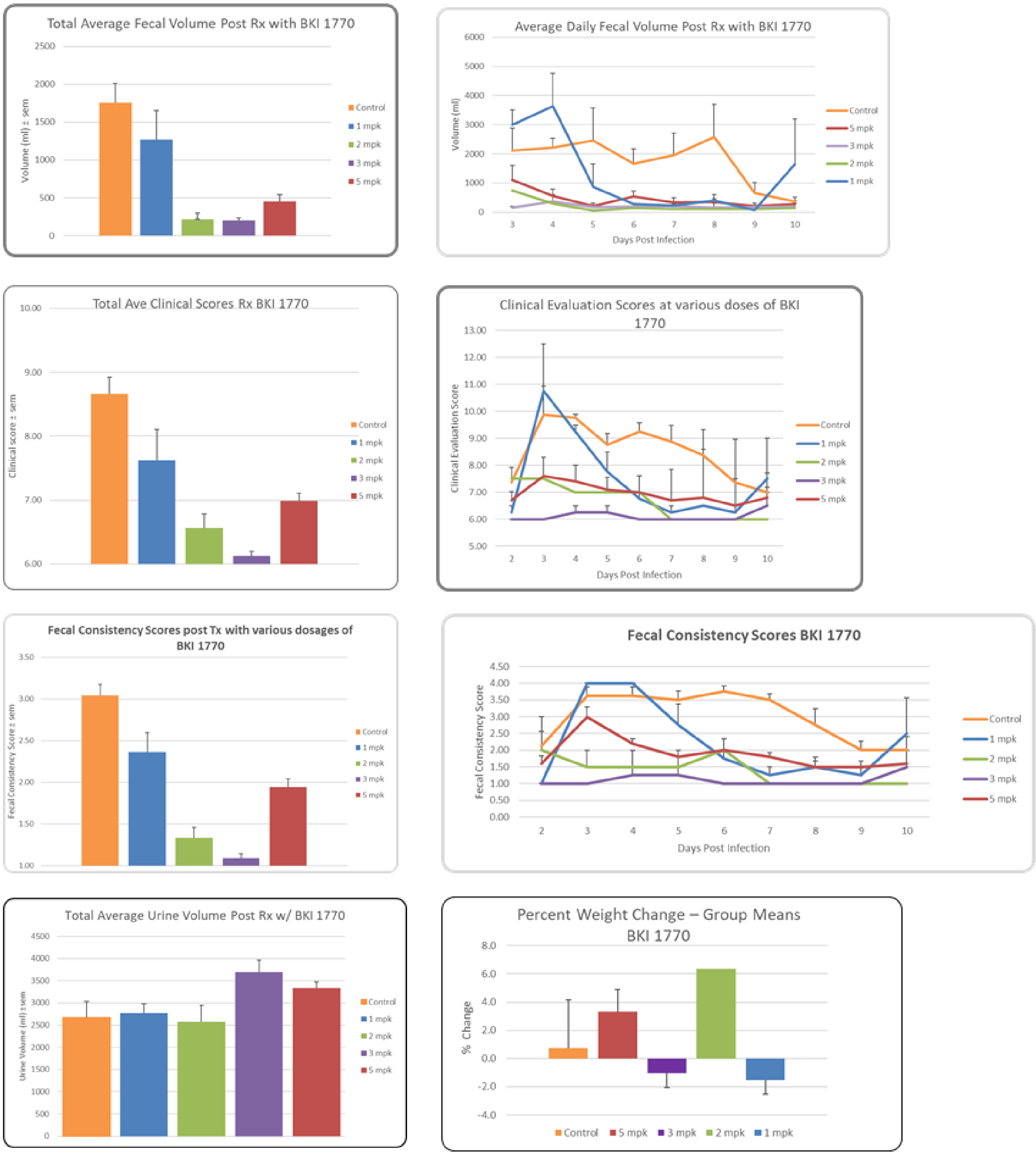

B.

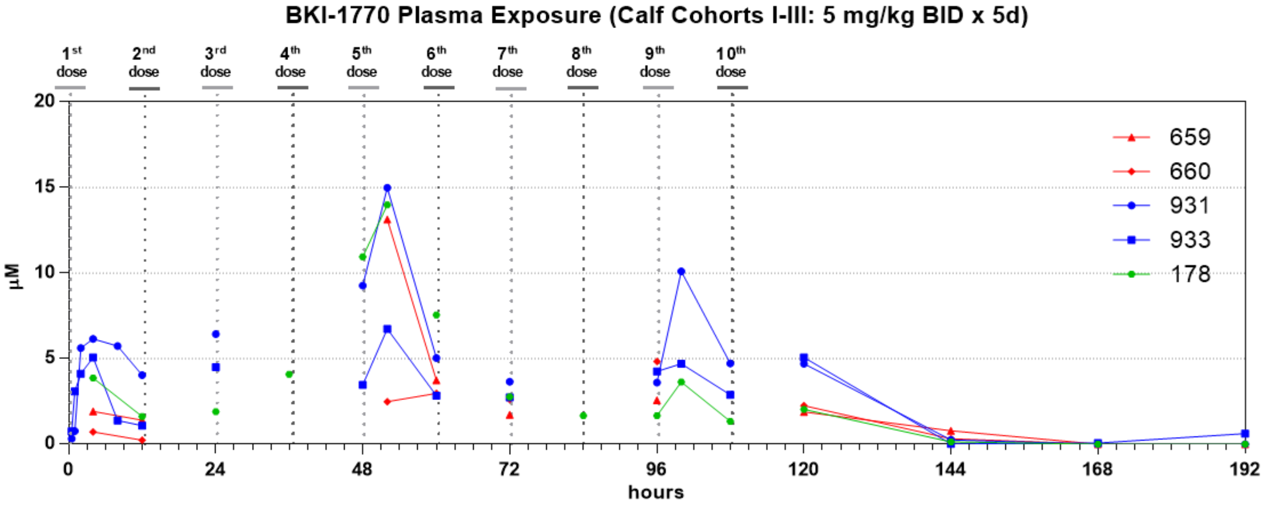
BKI-1770 Plasma Exposure (Calf Cohorts I-III: 5 mg/kg BID x 5d)
1st dose
2nd dose
3rd dose
4th dose
5th dose
6th dose
7th dose
8th dose
9th dose
10th dose
µM
hours
659
660
931
933
178

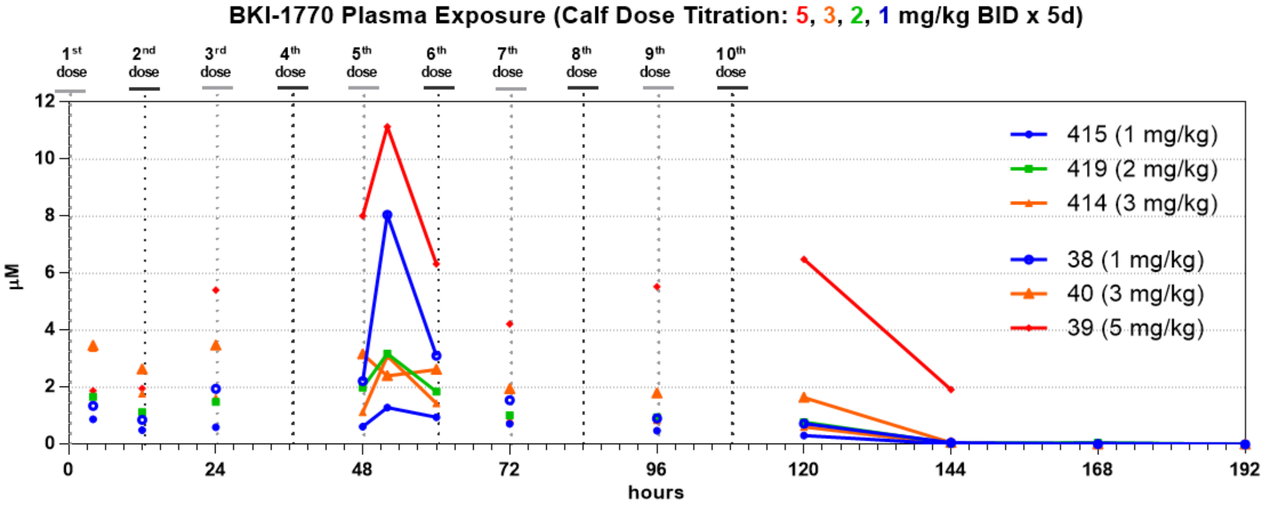
BKI-1770 Plasma Exposure (Calf Dose Titration: 5, 3, 2, 1 mg/kg BID x 5d)
1st dose
2nd dose
3rd dose
4th dose
5th dose
6th dose
7th dose
8th dose
9th dose
10th dose
µM
hours
415 (1 mg/kg)
419 (2 mg/kg)
414 (3 mg/kg)
38 (1 mg/kg)
40 (3 mg/kg)
39 (5 mg/kg)

Supplemental Figure 4. BKI-1841 dosed at 5, 2, and 1 mg/kg BID for 3 days and 2 mg/kg QD for 4 days in neonatal calves infected with *C. parvum*. (A) Urine volumes, clinical evaluation scores, fecal volume, fecal consistency, and weight change in all calves compared to untreated controls. (B) Plasma concentrations of BKI-1841 during dosing.

A.

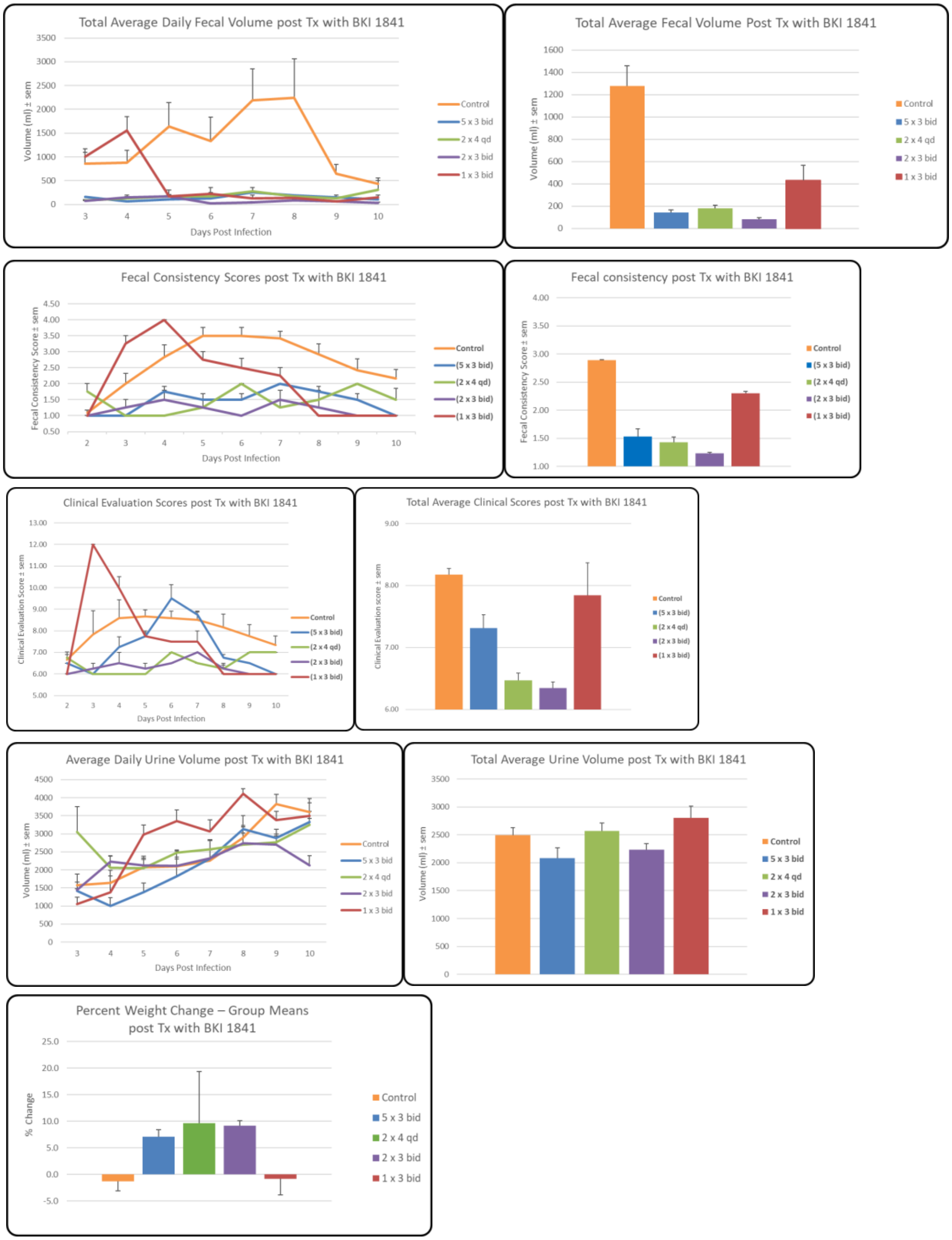

B.

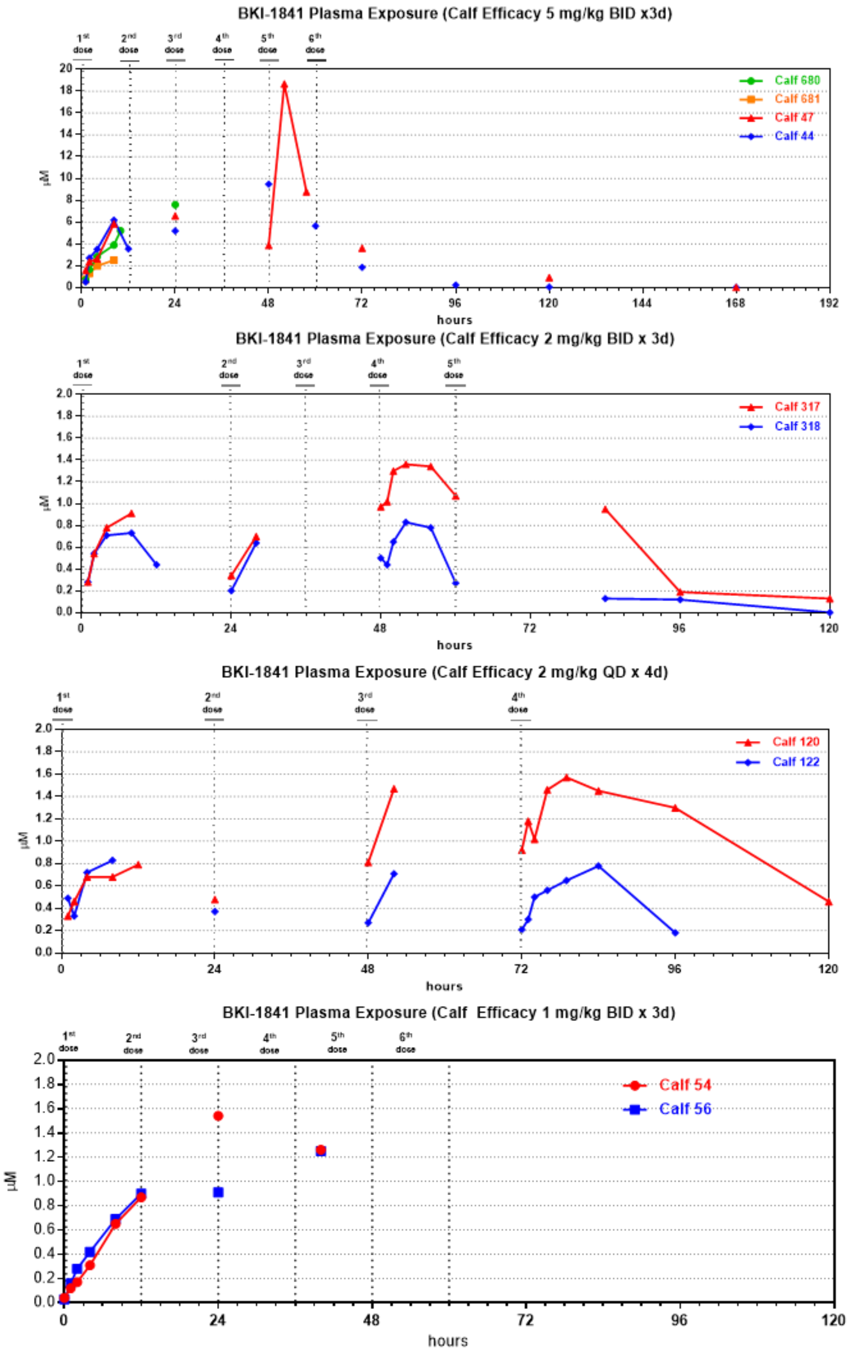
BKI-1841 Plasma Exposure (Calf Efficacy 5 mg/kg BID x3d)
Calf 680
Calf 681
Calf 47
Calf 44
μM
hours
BKI-1841 Plasma Exposure (Calf Efficacy 2 mg/kg BID x 3d)
Calf 317
Calf 318
μM
hours
BKI-1841 Plasma Exposure (Calf Efficacy 2 mg/kg QD x 4d)
Calf 120
Calf 122
μM
hours
BKI-1841 Plasma Exposure (Calf Efficacy 1 mg/kg BID x 3d)
Calf 54
Calf 56
μM
hours

Supplemental Figure 5. (A) Bone toxicity observed in hind limbs of calves treated with BKI-1770. (B) Changes to the epiphyseal growth plate in calves treated with BKI-1770. (C) Hyperflexion of the limbs seen as dropped pasterns observed in calves dosed with BKI-1841.

A.

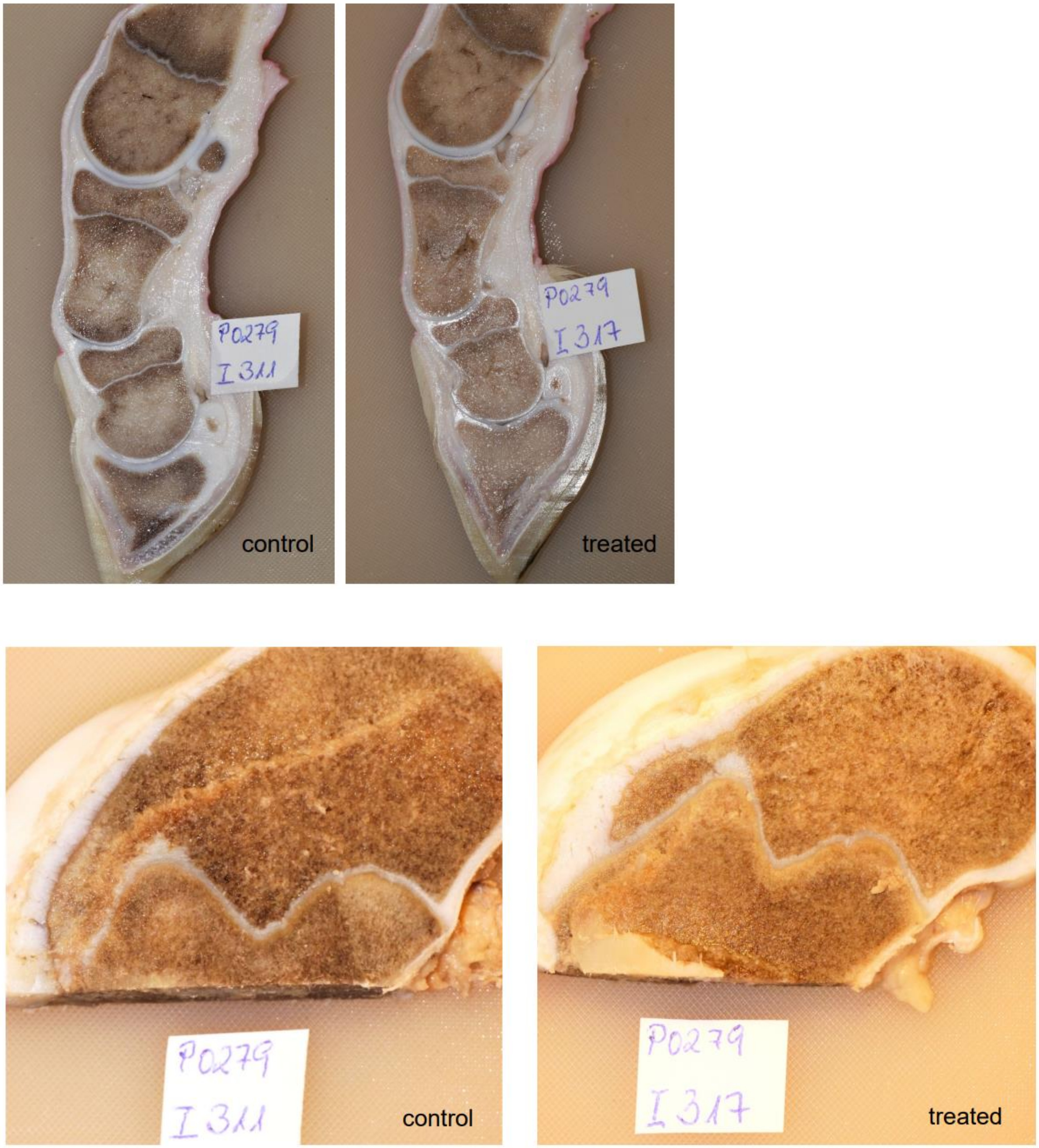

B.

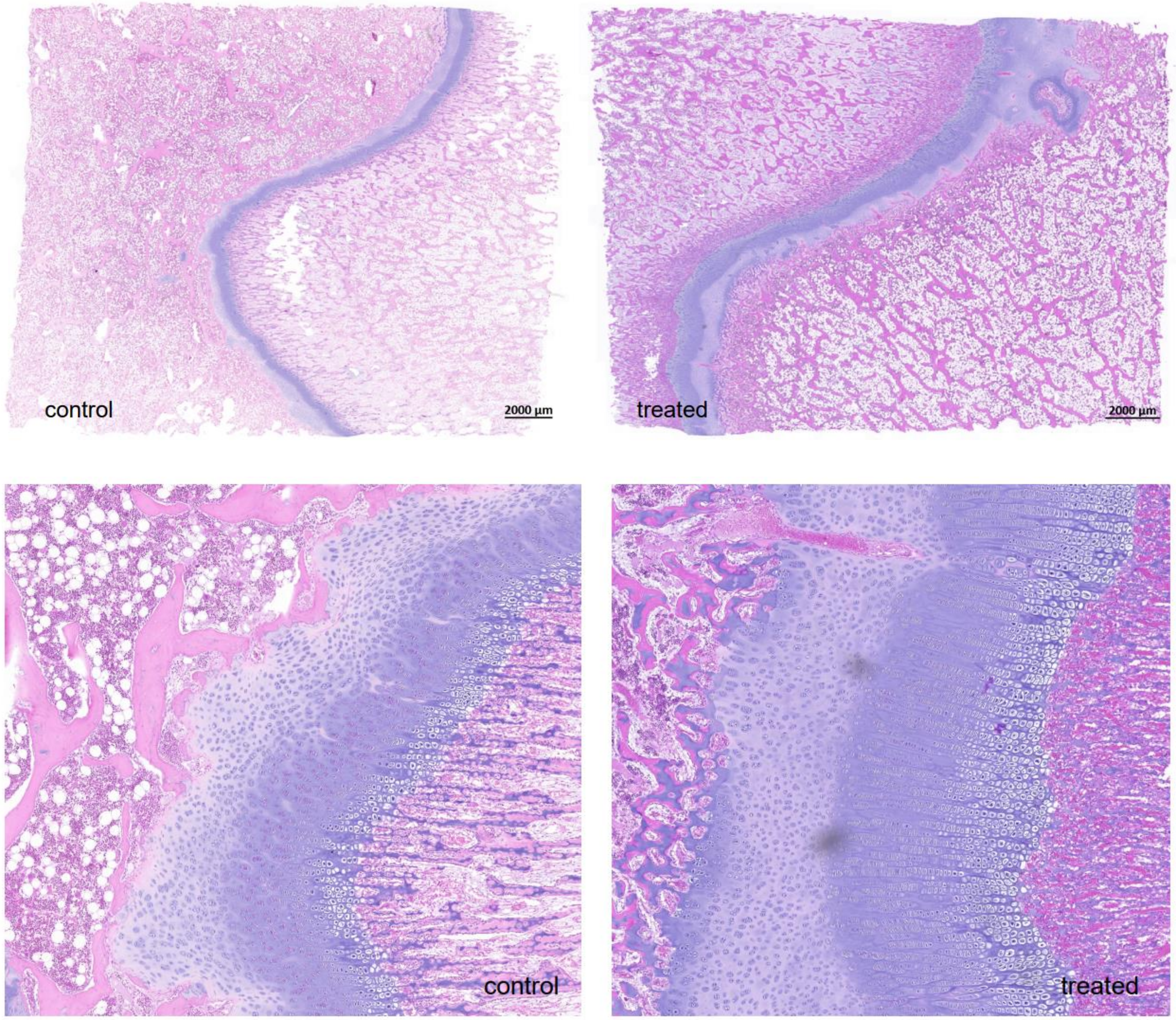

C.

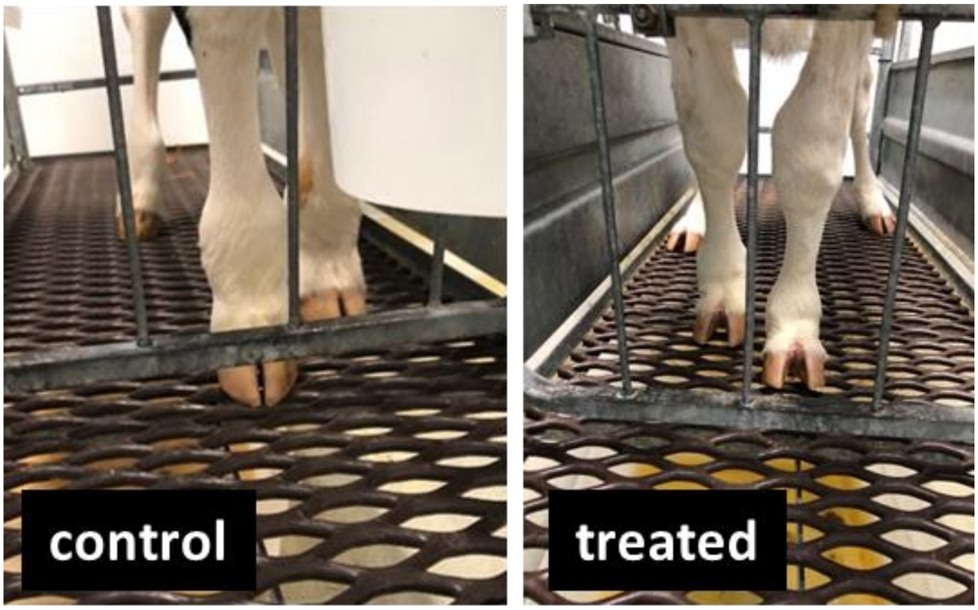

Supplemental Figure 6. (A) BKI-1770 dosed at 5 mg/kg BID for 5 and 8 days in neonatal piglets infected with *C. hominis*. Rectal swabs were processed for oocyst count and DNA measurement. The plots represent mean ± SEM. Wilcoxon matched-pairs signed rank test was conducted using GraphPad Prism 7.03. Body weight was measured daily. The plot represents mean ± SEM. Two-way ANOVA with Tukey's multiple comparisons test was conducted using GraphPad Prism 7.03. No significance between groups was observed for body weight. (B) Plasma and rectal swab concentrations of BKI-1770 during dosing.

A.

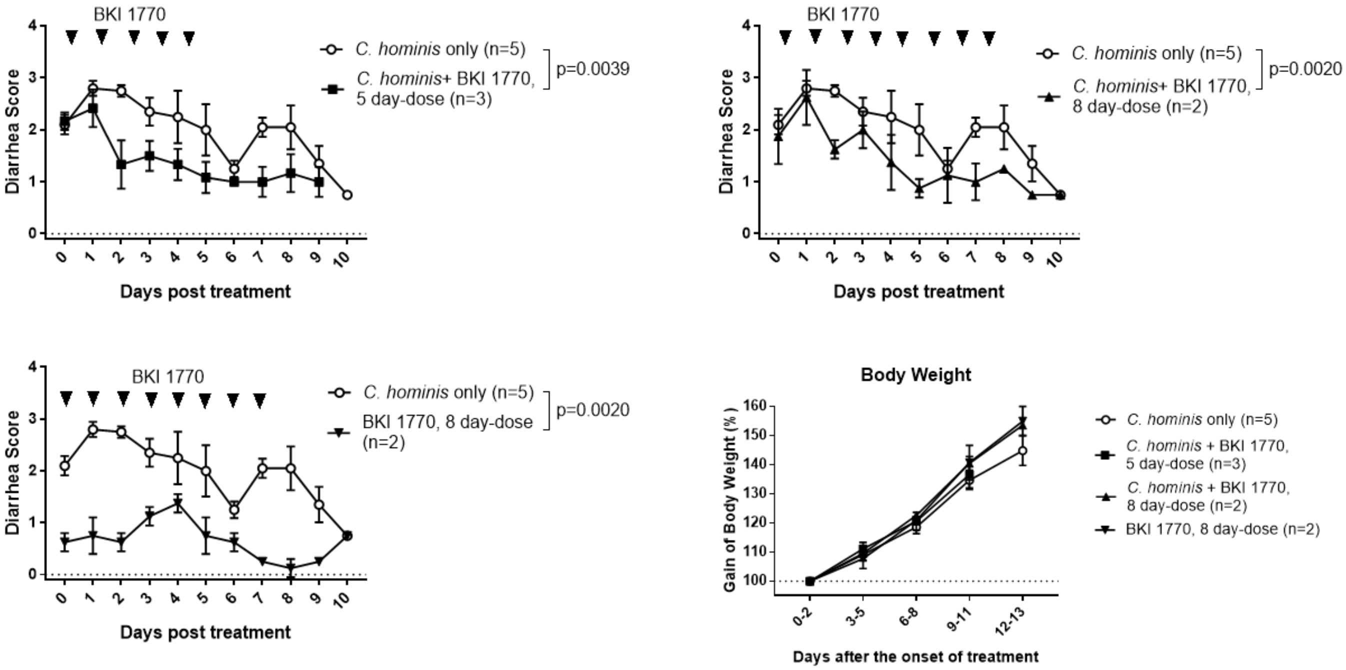

B.

Plasma concentration (μM)				
Time (h)	Piglet 4	Piglet 6	Piglet 7	Piglet 8
0	0.00	0.00	0.00	0.00
2	1.17	0.51	1.09	1.11
96	0.50	0.71	1.15	0.63
98	0.95	1.39	1.55	4.10
168	0.20	0.40	0.58	0.40
170	0.74	1.23	1.36	0.43
240	0.11	0.03		0.04
312	0.09	0.03	0.03	0.03

Rectal swab concentration (μM)			
Time (h)	Piglet 4	Piglet 6	Piglet 8
0	0.00	0.00	0.00
24	0.21	1.34	0.73
48	0.71	0.68	1.83
72	0.92	1.46	1.76
96	0.61	0.80	2.32
120	0.52	0.45	1.16
144	0.54	0.49	0.00
168	0.63	0.28	1.99
192	0.34	0.56	0.14
216	0.06	0.06	0.18
240	0.14	0.00	0.19
264	0.02	0.02	0.03
288	0.03	0.01	0.02
312	0.01	0.00	0.01

Supplemental Figure 7. Mouse Locomotor Activity Box results. Mice were dosed with BKI-1770, BKI-1708, and BKI-1841 at 150 mg/kg QD for 5 days.

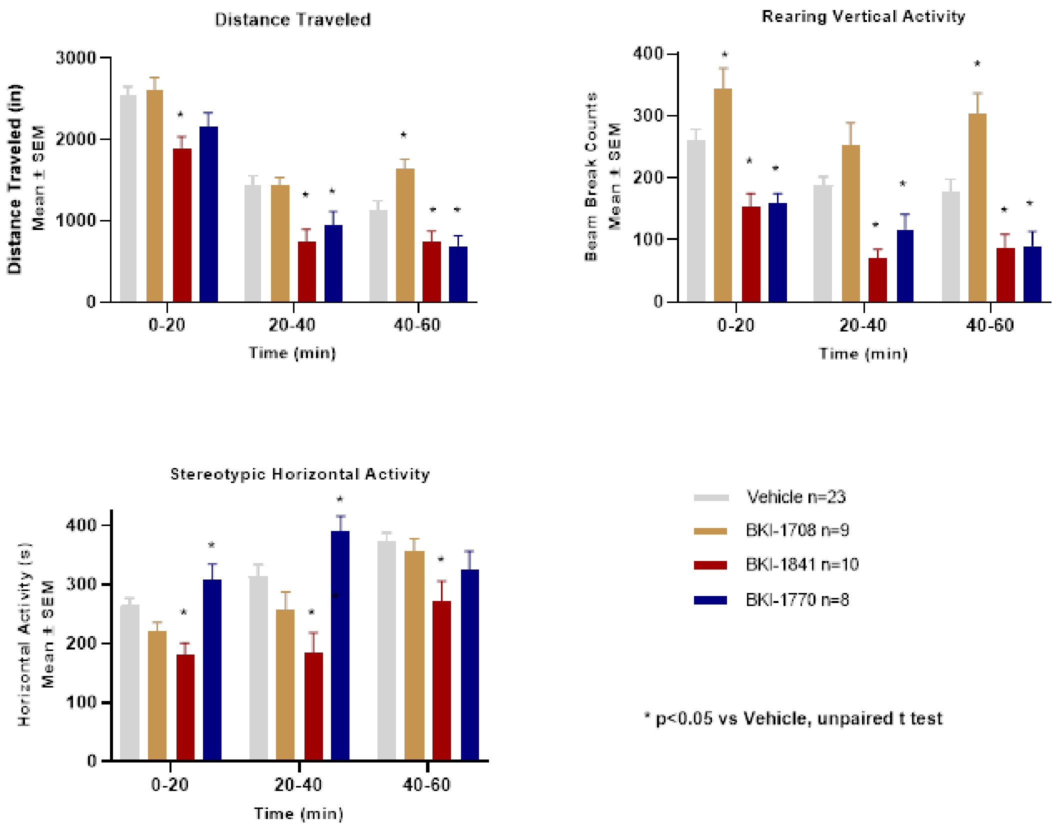

Supplemental Table 1. Rat cardiotoxicity results from intravenous dosing of BKI-1770 and BKI-1841. MAP – mean arterial pressure, HR – heart rate, dP/dt@50(%) – left ventricular contractility.

BKI	Dose (mg/kg)	MAP (%)	HR (%)	dP/dt@50 (%)
BKI-1770	3	-7	8	0
	10	5	8	9
	30	-7	3	8
BKI-1841	3	1	5	3
	10	3	3	4
	30	2	0	11

**Significant changes considered to be >15%*

Supplemental Table 2. Dog cardiotoxicity results from intravenous dosing of BKI-1770 at 0.02 mL/kg/min. MAP – mean arterial pressure, HR – heart rate, dP/dt@50(%) – left ventricular contractility, SVR – systemic vascular resistance, CO - cardiac output, QTcV – QT interval corrected using Vna der Water formula, QRS – ventricular depolarization time, PR – AV conduction time.

Time (min)	MAP (%)	HR (%)	dp/dt@50 (%)	SVR (%)	CO (%)	QTcV (ms)	QRS (ms)	PR (ms)
15	2	4	1	2	0	4	0	-3
30	2	5	0	-1	4	1	0	0
45	2	8	1	-2	3	1	-1	-1
60	2	9	-1	2	-1	-1	-2	-1
75	0	9	-2	1	-1	-3	-2	-1
90	-1	10	-6	8	-9	-2	-2	0
105	-2	9	-7	2	-4	-1	-3	0
120	-2	10	-7	4	-5	0	-2	-1
135	-3	11	-7	7	-8	-2	-1	-1
150	-5	13	-7	4	-7	-1	-1	-2

**Significant changes considered to be >15%*

REFERENCES

1. Huang W, Hulverson MA, Choi R, Arnold SLM, Zhang Z, McCloskey MC, Whitman GR, Hackman RC, Rivas KL, Barrett LK, Ojo KK, Van Voorhis WC, Fan E. 2019. Development of 5-Aminopyrazole-4-carboxamide-based Bumped-Kinase Inhibitors for Cryptosporidiosis Therapy. J Med Chem 62:3135-3146.
